# Comparison of Convolutional Neural Networks and Transformers for the Classification of Images of COVID-19, Pneumonia and Healthy Individuals as Observed with Computed Tomography

**DOI:** 10.3390/jimaging8090237

**Published:** 2022-09-01

**Authors:** Azucena Ascencio-Cabral, Constantino Carlos Reyes-Aldasoro

**Affiliations:** giCentre, Department of Computer Science, School of Science and Technology, City, University of London, London EC1V 0HB, UK

**Keywords:** deep neural networks, COVID-19, weighted cross entropy, bootstrap, Friedman–Nemenyi tests, transformer

## Abstract

In this work, the performance of five deep learning architectures in classifying COVID-19 in a multi-class set-up is evaluated. The classifiers were built on pretrained ResNet-50, ResNet-50r (with kernel size 5×5 in the first convolutional layer), DenseNet-121, MobileNet-v3 and the state-of-the-art CaiT-24-XXS-224 (CaiT) transformer. The cross entropy and weighted cross entropy were minimised with Adam and AdamW. In total, 20 experiments were conducted with 10 repetitions and obtained the following metrics: accuracy (*Acc*), balanced accuracy (*BA*), *F*_1_ and *F*_2_ from the general *F*${}_{\beta }$ macro score, Matthew’s Correlation Coefficient (*MCC*), sensitivity (*Sens*) and specificity (*Spec*) followed by bootstrapping. The performance of the classifiers was compared by using the Friedman–Nemenyi test. The results show that less complex architectures such as ResNet-50, ResNet-50r and DenseNet-121 were able to achieve better generalization with rankings of 1.53, 1.71 and 3.05 for the Matthew Correlation Coefficient, respectively, while MobileNet-v3 and CaiT obtained rankings of 3.72 and 5.0, respectively.

## 1. Introduction

Coronavirus 2019 (COVID-19) is an infectious disease caused by the Severe acute respiratory syndrome coronavirus 2 (SARS-CoV-2) [1], which has lead to a pandemic with millions of cases and deaths confirmed all over the world. The outbreak has triggered not only a health crisis but has also had a severe psychological, social and economic impact worldwide [2,3]. Attempts to improve the diagnosis, contain, and reduce the spread of the disease, has led COVID-19 to become one of the most researched topics in the world. At the time of writing (June 2022), PubMed reported 269,111 COVID-19-related entries (https://pubmed.ncbi.nlm.nih.gov/?term=covid-19 (accessed on 1 June 2022)), a significant number accumulated in just two years as prior to 2019 there are only 56 entries. Diagnosis of COVID-19 with Reverse Transcriptase Polymerase Chain Reaction (RT-PCR) tests is widespread; however, its sensitivity is only moderate [4,5,6,7,8]. For this reason, medical imaging diagnosis with radiographs and Computed Tomography (CT) has been widely used [9,10,11] as it is generally considered more reliable for the identification of COVID-19 hallmarks, which include ground glass opacity with or without consolidation in the posterior and peripheral lung, linear opacity, “crazy-paving” pattern, “reversed halo” sign and vascular enlargement in the lungs [12,13,14,15].

Deep Learning (DL) is a sub-field of Artificial Intelligence (AI) that enables algorithms to automatically extract features from data, *learn* patterns and characteristics, and generate predictions on unseen data.

Some principles and ideas behind DL and AI have been known for decades, e.g., the WISARD architecture from 1984 [16] or the Self Organising Maps from 1982 [17]). Interestingly, discussions about the hype or reality of Neural Computing [18] and how difficult Artificial Intelligence really is [19], have remained. With the addition of a large number of multiple processing layers, thus the use of the term deep, the availability of large data sets and the increase computational power, these techniques have recently shown excellent results in many areas [20]. These multiple layers allow a large number of nonlinear modules to convert the representation of the input data, which can be an image or text, to a more abstract representation [21]. The breakthrough of deep learning is sometimes related to the outstanding results presented in the classification of the ImageNet Large Scale Visual Recognition Challenge (ILSVRC) [22]. In medical applications, deep learning architectures have provided excellent results, for instance, in the classification of skin cancer images [23].

Recent studies on image analysis of radiographs and CT scans have shown that DL-based methods are capable of detecting, quantifying and monitoring COVID-19 with high accuracy [24,25,26,27,28,29]. For conventional radiographs or 2D X-ray images, deep learning approaches have combined Convolutional Neural Networks with Long Short Time Memory (CNN-LSTM) models [30], Genetic Adversarial Networks with Long Short Time Memory [31], or self-augmentation mechanisms [11], with the objective to distinguish between different cases such as healthy against disease, COVID against pneumonia, etc. Some studies have focused on the segmentation of regions of interest within the lung region [32,33,34] using modifications of the popular U-Net architecture [35]. The present study focused on classification of the images and not in segmentations, in part because there was no ground truth available, but also because the focus was the methodology to compare the classification with non-parametric statistics. CT scanners, as well as Magnetic Resonance Imaging and other medical imaging devices can generate volumetric data, which in some cases can provide better results than analysis on a per-slice basis [36]. As such, some COVID studies have focused on the volumetric analysis of the data. For instance, Bougourzi analysed the percentage of the COVID infection to infer the state of patients (e.g., Normal, Moderate, Severe, etc.) [37,38,39]. It is also possible to combine slice-level decisions with tools such as Long Short Term Memory models [40]. When data other than images is present, i.e., medical notes, electronic health records or audio recordings, it is possible to perform multi-modal diagnosis [41,42,43]. However, it is not always the case that researchers have access to multi-modal data and thus thorough evaluation of a single type of data is important. Ensemble techniques, in which several deep learning models are trained and a decision is taken based on votes from all the models are popular [44,45,46] and can provide good results. However, the energy consumption of training many models, several of which may provide suboptimal results should be considered in the present world where carbon footprint of computational processes is not negligible [47]. For further information about approaches to COVID, the reader is referred to recent review papers, e.g., [48,49,50,51]. Many of the reported COVID studies consist mainly on fine-tuning pre-trained convolutional networks [24,28,52,53], or ensembles of methods together with optimization techniques [54,55]. Although these studies have shown very promising results, a large proportion of them do not provide sufficient information about the how and from where the data are sourced, how the data are handled and pre-processed if at all, the training configurations or statistical grounds to support why a proposed model is significantly better than another and studies have shown that the limitations of studies still present little value in clinical settings [56].

To address these issues, in this work, 20 experiments were built based on five DL architectures: CaiT-24-XXS-224, DenseNet-121, MobileNet-v3-large, ResNet-50 and ResNet-50r. The first selection was the ResNet architectures, which have had a major influence on the design of deep neural convolutional and sequential networks followed by DenseNet and MobileNet. These are based on convolutions and the latter is designed for mobile applications. Meanwhile CaiT is a state-of-the-art transformer that does not use convolutions. Each of these DL architectures were developed in intervals of roughly two years from 2015 to 2021. Table 1 outlines the parameters, the inference time required in GPU/CPU (running in GPU), the operations utilising more resources when processing images and the year in which each of the architecture was published. Whilst there are numerous options of architectures, for the present work, it was considered that the choice of architectures were representative of the fast developments in artificial neural networks for application in computer vision.

For each architecture, two loss functions (cross entropy and weighted cross entropy) and two optimisers (Adam and AdamW) were applied and the architectures were evaluated with seven metrics. In addition, the results for each metric were bootstrapped for 1000 cycles and their prediction power was further compared with non-parametric statistics for robustness in different scenarios. Thus the main contributions of this work are summarised as follows:

- A well-structured experimental setup for the evaluation and unbiased comparison of the performance of five representative deep learning architectures (CaiT-24-XXS-224, DesNet-121, MobileNet-v3-large, ResNet-50 and ResNet-50r) for the classification of COVID-19 as observed with Computed Tomography was proposed.
- The ResNet-50r architecture, which is based on ResNet-50 but the convolutional layer (Conv1) with filters of size 5×5, was used to observe the effect of the kernels size (filters) on the classification of COVID-19.
- Bootstrapping technique was applied to derive a very large number of samples, which will compensate for any cases of outliers for non-normal distributed data.
- The results of each deep network architecture and experiment with different optimisers and loss functions were compared using non-parametric statistical comparison of the performance of deep network architectures.
- The results show that less resource-demanding networks can outperform more complex architectures. This is a significant consideration related to the energy consumption necessary to train deep learning architectures in the light of the current climate emergency, and given the climate emergency of the present world [57,58,59].

## 2. Materials and Methods

### 2.1. Data Collection

**Figure 1:**
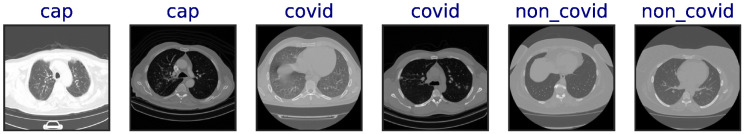
Illustration of six representative Computed Tomography (CT) images of the three different classes: community-acquired pneumonia (CAP), COVID and non-COVID.

To build the classification dataset, data from two public repositories was collated. The first dataset was sourced from Kaggle [60,61]. This dataset comprises a multi-nation collection of curated COVID-19 CT scans from 7 public sources [62]. This dataset contained 7593 curated images from 466 patients that were diagnosed with COVID-19, 6893 images from 604 patients that were considered as healthy with normal lungs, and 2618 images from 60 patients diagnosed with community-acquired pneumonia (CAP). The second dataset was sourced from Mendeley Data [63] and contained COVID-19 and common pneumonia (CP) CT scans. From this dataset only used 328 CP images which were merged with the CAP images were used. Figure 1 shows some representative images of COVID-19, CAP and Non-COVID (normal lungs) from the collated dataset. The dataset was split by class stratification to ensure all subsets had the same number of images from the minority class “CAP”. The split ratios were 80:10:10 for training (13,945 images), validation (1743 images) and test (1744 images), respectively. Table 2 shows the number class instances per subset.

### 2.2. Deep Learning Architectures

To build the classification experiments (models) four pre-trained out-of-the-box deep convolutional architectures with different levels of complexity and one transformer were proposed. Next, the prediction power of CaiT-24-XXS-224, DenseNet-121, MobileNet-v3-large, ResNet-50, ResNet-50r on the collated multi-class COVID-19 dataset were evaluated and compared.

ResNet-50 is a 50-layer deep convolutional architecture that features 3-layer skip connections (bottleneck block). The skip connections enable the network to copy activation from bottleneck block to bottleneck block [64]. To built ResNet-50r the kernels from the first convolutional layer (Conv1d) of ResNet-50 were resized from 7 × 7 to 5 × 5. The term ‘r’ was used to indicated that the network had resized kernels. DenseNet-121 (121 layers deep) consists of multiple dense blocks (small convolutional layers, batch normalisation and ReLU activation) and transition layers. Each layer in a dense block is forward-connected to every other layer by using concatenation shortcuts [65]. A MobileNet-v3-large (MobileNet-v3) is a convolutional neural network designed for mobile and embedded vision applications. It is based on a streamlined architecture that uses depth-wise separable convolutions. MobileNet-v3 has an efficient last stage at the end of the network that further reduced latency [66]. Transformers were initially used in the field of Natural Language Processing [67] and have been recently adapted for large scale image classification, demonstrating that convolutional networks are not strictly necessary for image processing. Class Attention in Image Transformers (CaiT) is a transformer for computer vision applications created to optimise the performance of the transformers when they have large number of layers [68]. CaiT-24-XXS-224, indicates that the architecture has a depth of 24 class attention layers, working dimensionality of 192 and was trained at resolution 224. The parameters of each architecture are shown in Table 1.

### 2.3. Experimental Setup

The experimental setup for this work is described in Table 3. The experiments (classifiers) were designed by combining three factors: neural network architecture, the loss function (objective) and the optimiser to minimise the loss. To detect the impact of the class imbalance on the predictions, the weighted version of the cross entropy (wCE) loss function was minimised with Adam and AdamW. The wCE handles the class imbalance by penalising with higher cost (weight) misclassification of the minority class. The Adam optimiser (derived from adaptive moment estimation) is an optimiser in which the learning rate is adaptive and can handle sparse gradients on noisy problems [69]. AdamW is Adam with weight decay and the primary choice to train transformer models. Whilst there are many other options for optimisers such as adaptive gradient algorithm (Adagrad), stochastic gradient descent (SGD), SGD Momentum (SGDM), Root Mean Square Propagation (RMSprop), Adam and AdamW were selected due to fact that these adaptive gradient methods do not underperform momentum or gradient descent optimisers [70]. Adam is probably the most popular optimiser option. A search for “Adam optimiser” in Google Scholar (https://scholar.google.co.uk/scholar?q=adam+optimizer accessed on 4 August 2022) returned 103,000 entries. Equivalent searches returned fewer entries: Adagrad: 10,200, SGD: 36,300, SGDM: 1500, RMSProp: 20,700, AdamW: 5950. On the other hand, AdamW has shown to improve the performance of Adam by decoupling the weight decay and outperforming SGD Momentum in image classification tasks [71].

**Figure 2:**
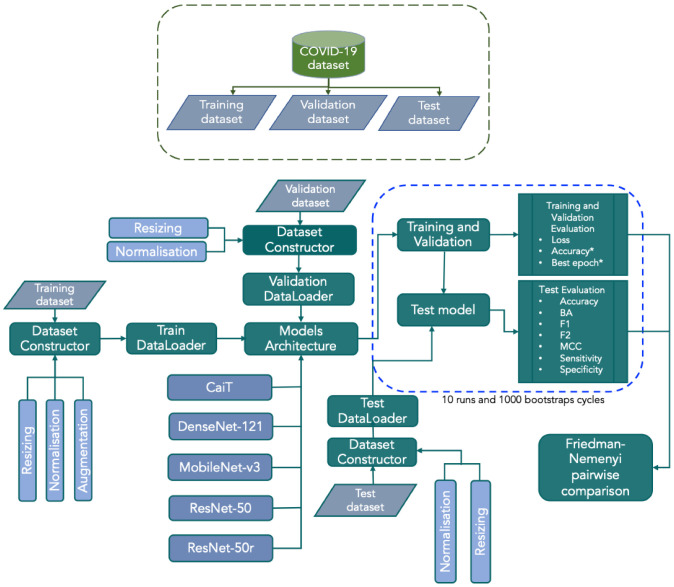
Graphical illustration of the pipeline steps used for the training, evaluation and comparison of the deep neural network models for the classification of COVID-19 in a multi-class setup used in this work. All outputs from test phased were bootstrapped. Training, validation and test were run 10 times. After this, the test and validation results were further bootstrapped for 1000 cycles. * Indicates the bootstrapped outputs from validation phase.

With these, 20 experiments were built and evaluated (5 × 2 × 2) by training CaiT, DenseNet, MobileNet-v3, ResNet-50, ResNet-50r to minimise CE and wCE with Adam or AdamW (Table 3). Figure 2 illustrates the pipeline approach to classification and comparison of the models in out experimental setup. All experiments were implemented in PyTorch Framework and run on google Colab Pro and Pro+. The code is available on an *as-is* basis on the following github repository: https://github.com/ace-aitech/COVID-19-classification (accessed on 4 August 2022).

### 2.4. Training and Validation

All networks architectures described in Section 2.2 were pre-trained on the ImageNet dataset. Thus, the images were normalised and resized to align them to the pre-trained setup. On-line random horizontal flips augmentations were also applied at training. The approach to training was transfer learning by fine-tuning the experiments for 8 epochs with a learning rate of $2\times 10^{-5}$ in batches of 8 images. Training and validation classification loss and accuracy were calculated after each epoch of training. The validation accuracy was the primary indicator on how well the classifiers were performing.

### 2.5. Performance Metrics for Evaluation

The training procedure outlined in Section 2.4 and the evaluation on the test dataset was repeated 10 times per experiment. To make an unbiased comparison of the classifiers, the weights from the last epoch of training for evaluation on the test set were used. For this study, True/False Positives/Negatives (TP/TN/FP/FN) were defined by the correct or incorrect prediction of the class for the whole image. Accuracy (Acc), Balanced Accuracy (BA), *F*_1_ and *F*_2_ from the general *F*${}_{\beta }$ macro score, Matthew’s Correlation Coefficient (MCC), Sensitivity (Sens) and Specificity (Spec) were used to evaluate the performance of models by experiment and network. These seven metrics are defined as follows:

(1) $$Acc=\frac{TP+TN}{\left(TP+TN+FP+FN\right)},$$

(2) $$BA=\frac{\frac{TP}{\left(TP+FN\right)}+\frac{TN}{\left(TN+FP\right)}}{2},$$

(3) $$F_{\beta }=\frac{(1+\beta^{2})TP}{\left(\left(1+\beta^{2}\right)TP+\beta FP+FN\right)},$$

(4) $$F_{1}=\frac{2TP}{\left(2TP+FP+FN\right)},$$

(5) $$F_{2}=\frac{(5)TP}{\left((5)TP+2FP+FN\right)},$$

(6) $$MCC=\frac{\left((TP\times TN)-(FP\times FN)\right)}{\left((TP+FP)+(TP+FN)+(TN+FP)+(TN+FN)\right)},$$

(7) $$Sens=\frac{TP}{\left(TP+FN\right)},$$

(8) $$Spec=\frac{TN}{\left(TN+FP\right)}.$$

Despite being widely used to evaluate the performance of classifiers, accuracy is biased towards the majority class in imbalanced datasets. On the other hand, Precision, Recall, *F*${}_{\beta }$ macro score and MCC have been widely used to overcome the imbalance problem [72]. The *BA* provides an average measure of how likely an instance of a class is correctly classified across different classes. It consists of the arithmetic mean of the recall of each class, so it is “balanced” because every class has the same weight and the same importance [73]. The macro *F*${}_{\beta }$ score is a weighted harmonic mean of the macro-precision and the macro-recall. For the multi-class setup, the *F*_1_ and *F*_2_ where β takes the values of 1 and 2, respectively, were used. *F*${}_{1}$ score weights all classes equally (recall and precision). [73] whereas *F*${}_{2}$ score weights twice the recall favouring it against precision. *F*${}_{2}$-score severely penalizes false negatives. The MCC is a measure of the correlation between the true and the predicted class. Moreover, it is regarded as a good indicator of total imbalanced of the prediction model. Recent work [72] demonstrated that the MCC is a well-suited metric for imbalanced multi-class domains. The sensitivity (or recall) is the number of true positive results divided by the number of all samples that should have been identified as positive. Specificity is the fraction of the true negatives divided by the total number of negatively classified instances.

### 2.6. Statistical Comparison

Non-parametric statistics do not require the distribution of the data to be known to make assumptions about them. Parametric comparison tests such ANOVA and MANOVA not only assume that samples come from a normal distribution but most importantly that all variables have equal variance (sphericity). For comparison of intelligent algorithms this cannot be assumed and can also have a detrimental impact on the post hoc test [74]. Therefore, non-parametric Friedman omnibus test [75] and Nemenyi post hoc pairwise comparison were used [76]. In this work, a holdout approach to evaluate the performance of the classifiers (experiments) by using the metrics defined in Section 2.5 was used. Bootstrapping is a non-parametric method that consists of sampling, with replacement, from a single original sample. This allows an approximation of sample distribution of statistics from original data [77]. To build the initial sample, the hold-out process was ran and evaluated 10 times per experiment. Then, 1000 bootstrap samples per experiment were generated and the average ranking and confidence interval (CI) for each of the evaluation metrics by network and experiment were calculated. Bootstrapped *BA* has been used to compare traditional machine learning classifiers with DL methods for the stress recognition in drivers [78]. Statistical difference amongst the performance of experiments (classifiers) was determined by using the Friedman test followed by the Nemenyi post hoc test at α = 0.05 for the Acc, BA, *F*${}_{1}$, *F*${}_{2}$, MCC, Sens and Spec. These tests have been used to compare the performance of time series classification algorithms for gravitational waves [79]. The Friedman test indicates whether the ranked classifiers are significantly different amongst themselves while the Nemenyi test applies pairwise comparison to the ranked classifiers [74,80]. The statistical tests were applied by using scipy and scikit-post hoc libraries.

## 3. Results and Discussion

### 3.1. Training, Test and Validation Accuracy

**Figure 3:**
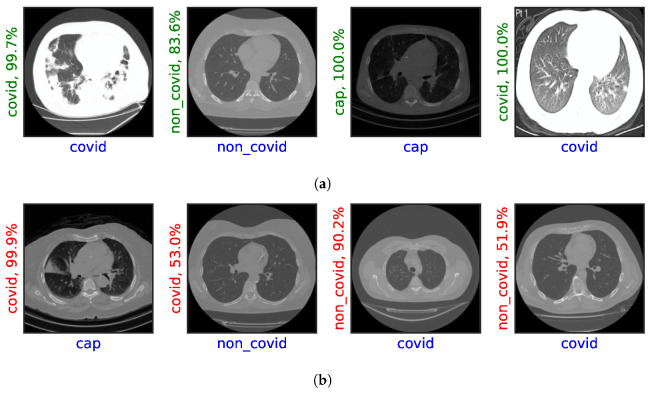
Illustration of results (horizontal label) with their prediction and probability score (vertical label). (**a**) Correct predictions. (**b**) Misclassifications.

Figure 3 illustrates the results of one experiment (Exp-13) comparing predicted and actual classes of representative images on the test set.

**Figure 4:**
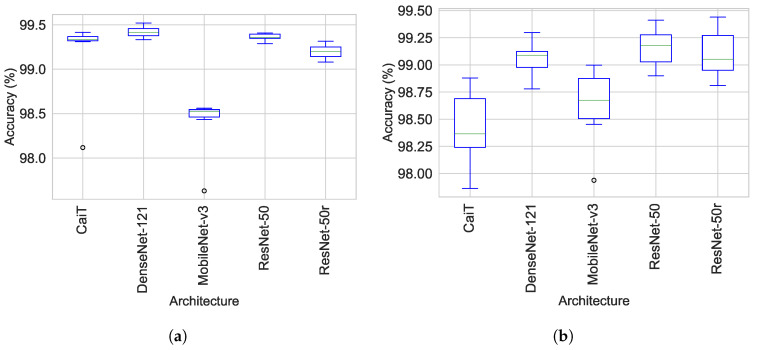

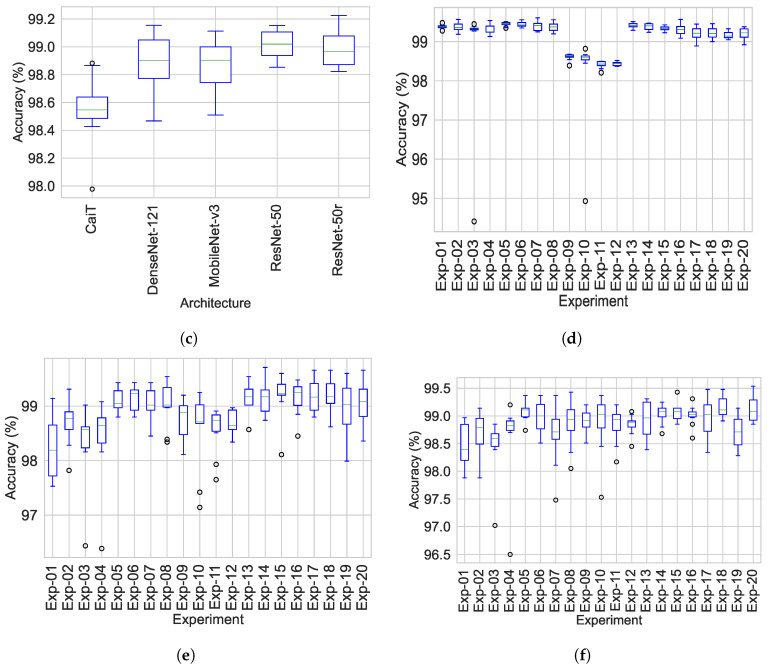
Train, validation and test accuracy before the bootstrapping cycle by network and experiment. (**a**) Train accuracy by architecture. (**b**) Validation accuracy by architecture. (**c**) Test accuracy by architecture. (**d**) Train accuracy by experiment. (**e**) Validation accuracy by experiment. (**f**) Test accuracy by experiment.

Accuracy and standard deviation for the training, validation and test sets by architecture and experiment are shown in Table 4 and Table 5, Figure 4. From the Figure 4 it can be seen that the ResNet-50 models achieved the highest accuracy during the validation and test phases. Table 4 shows that DenseNet models obtained the greatest accuracy of the five architectures during training. Conversely, CaiT based models showed the lowest accuracy on the validation and test stages. For the individual experiments the top three training accuracies were 99.45%, 99.44% and 99.42% for Exp-05, Exp-06 and Exp-13, respectively. The best validation accuracy was obtained for Experiments Exp-17 and Exp-18 (99.19% both of them) which are based on ResNet-50r, followed by Exp-13, Exp-15 which are built on ResNet-50. The top performers at the test phase were Exp-18, Exp-15 and Exp-05 (Table 5). At architecture level CaiT models showed the highest standard deviation for the three stages, in particular Exp-04 at training and test. It should be noted that in all phases and for all models, the average accuracy was greater than 98.0%.

### 3.2. Performance Metrics Prior to Bootstrapping

Table 6 summarises the performance metrics during the test phase by network prior to bootstrapping. The average performance of each network for all evaluation metrics was in the following descending order: ResNet-50, ResNet-50r, DesNet-121, MobileNet-v3 and CaiT (Figure 4). The five networks reached an average performance of over 98% for the 7 evaluation metrics, except for CaiT, which showed an average MCC of 97.64%. ResNet-50 achieved values over 99.0% in six of the seven evaluation metrics, followed by ResNet-50r which hit the the highest *F*${}_{1}$ and *F*${}_{2}$ scores and the same average performance than ResNet-50 in Sens and Spec. It was observed that the BA, $F_{1}$, $F_{2}$ and *Sens* presented very close values to each other when evaluating the performance of the architectures (Table 6). The *MCC* suggests that there is high correlation among the predictions and their real class and reflects the impact of the class imbalance [81]. In addition, from the metrics by experiment it was noted that, Exp-18 and Exp-20 obtained the highest values from all experiments for all metrics followed by Exp-05 and Exp-15 (Table 7). Exp-03 and Exp-04 showed the largest standard deviation in three and four of the evaluation metrics, respectively.

### 3.3. Ranking and Confidence Intervals Post-Bootstrapping

Table 8, Table 9, Table 10 and Table 11 summarise the medians, ranking and confidence intervals with α = 0.05 of the architectures and experiments by metric. The ranks of the networks from the best to poorest performance was as follows: ResNet-50, ResNet-50r, DenseNet-121, MobileNet-v3 and CaiT (Table 8). ResNet-50 models outperformed the other architecture models in 5 of the 7 evaluation metrics (*Acc*, *BA*, *MCC*, *Sens* and *Spec*). ResNet-50r surpassed ResNet-50 in the *F*_1_ and *F*_2_ scores. It also obtained the second best rank for the rest of metrics. Although the medians obtained for each metric by network were almost identical to their respective mean prior to bootstrapping, the confidence intervals were tighter (Table 6, Table 7, Table 8, Table 9, Table 10 and Table 11). In general, all networks showed wider CI for specificity than for sensitivity (Table 9). The *MCC* showed lower confidence bounds for all networks. CaiT confidence intervals by architecture were the lowest ranging from 97.36% to 97.88%. Models based on ResNet-50 and ResNet-50r performance lower bounds were greater than 99.0% in five of the seven metrics and over 98.0% for the *MCC* and accuracy. The ranking of the bootstrap samples by experiments for each metric is shown in Table 10. The top ranks in descending were achieved by Exp-18, Exp-20, Exp-05 and Exp-15. ResNet-50r based experiments outperformed all models metrics when trained to minimise the loss function with AdamW. Exp-18 showed the highest rank in all performance metrics except for the *MCC* where it ranked negligibly lower than Exp-20. The opposite effect was observed when training ResNet-50r to minimise the wCE with Adam (Exp-19). Exp-19 ranked the lowest for experiments based on ResNet-50r. The results suggests that the size of the kernel in ResNet-50r have a positive effect on the performance of Exp-18 and Exp-20 which are the modified versions of Exp-14 and Exp-16. Furthermore, Exp-15 and Exp-13 were the highest and lowest ranks for experiments built in ResNet-50. This effect might be due to the loss function that each of these two experiments optimised (wCE and CE, respectively). DenseNet-121 and MobileNet-v3 models performed better when minimising the CE loss function (Exp-05, Exp-06, Exp-09 and Exp-10). In contrast, experiments built on ResNet-50 perform better when minimising the wCE loss function. Further more, experiments built on CaiT and ResNet-50r performed their best when optimising with AdamW (Exp-02, Exp-04, Exp-18 and Exp-20). The lowest performance boundaries were 96.96% and 96.89% for the *MCC* (Table 11, Exp-03 and Exp-04).

### 3.4. Maximum Training Epochs

**Figure 5:**
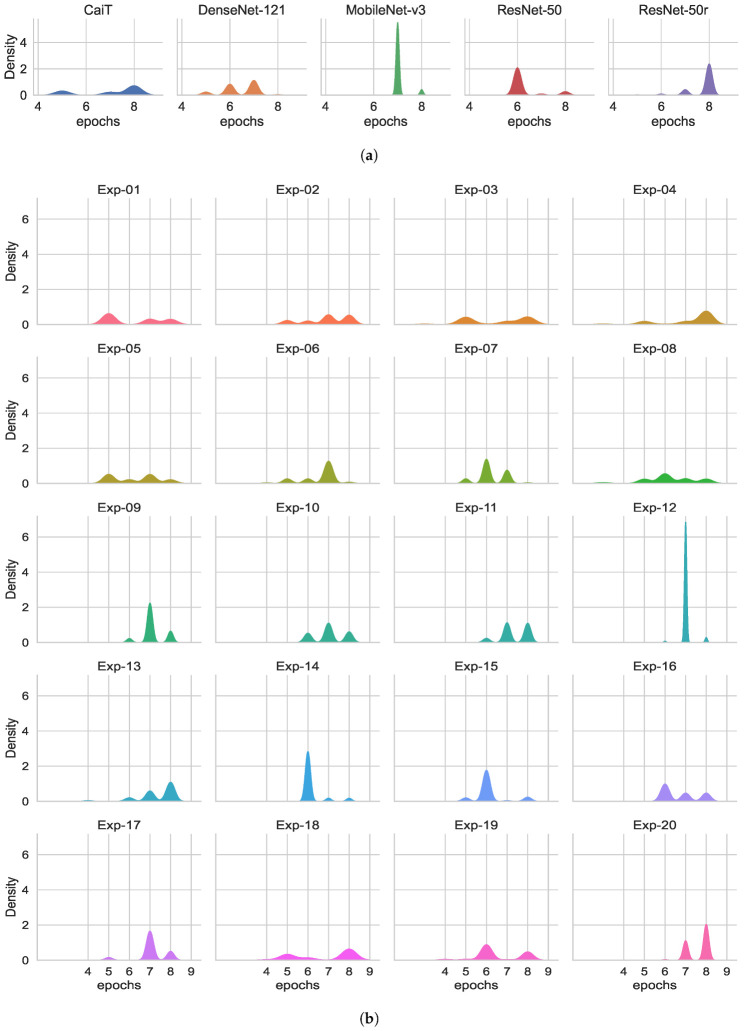
Epochs required to reach the maximum validation accuracy. (**a**) Epochs by architecture. (**b**) Epochs by experiment (CaiT experiments 1–4, DenseNet experiments 5–8, MobileNet experiments 9–12, ResNet-50 experiments 13–17, ResNet-50r experiments 17–20).

**Figure 6:**
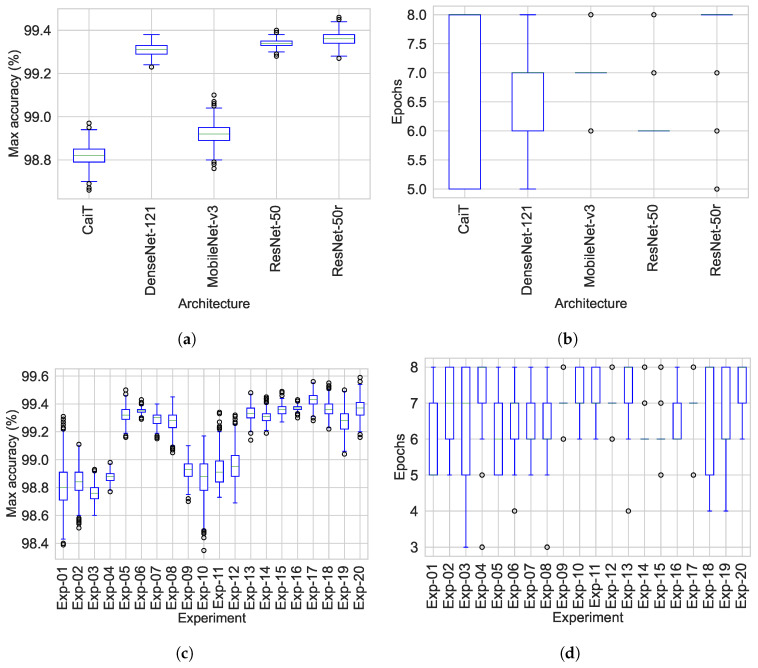
Maximum training accuracy and training epochs to reach the maximum accuracy after 1000 bootstrap cycles. (**a**) Max accuracy by architecture. (**b**) Max accuracy by experiment. (**c**) Epochs required to reach the maximum accuracy by architecture. (**d**) Epochs required to reach the maximum accuracy by experiment. CaiT experiments 1–4, DenseNet experiments 5–8, MobileNet experiments 9–12, ResNet-50 experiments 13–17, ResNet-50r experiments 17–20.

The training of the networks has a critical effect on the evaluation of the models on unseen data (generalisation performance). To have an insight into the number of training cycles required for each of the classifiers to achieve their best validation accuracy, the number of epochs at which each experiment obtained the highest validation accuracy and the accuracy achieved was recorded. Following this, 1000 bootstrap samples for the maximum validation accuracy and the number epochs required to reach the maximum were simultaneously obtained. Table 12 and Table 13 provide the ranking, the median of maximum accuracy and the number of epochs required to reach the maximum accuracy during validation by architecture and experiment. Figure 5 and Figure 6 show the medians and distribution of the data for both the maximum accuracy by architecture and experiment. The best ranks for the number of epochs were given to models based on ResNet-50 and Dense-Net-121 which required six and seven rounds of epochs training, respectively. Conversely, ResNet-50r and CaiT mostly required to train for 8 epochs to reach their best performance. The information in Table 1 and Table 12 provides a wider overview on how the GPU/CPU/Memory resources were utilised and their impact on training on the training and validation process. The operation that required more memory for CaiT was matrix multiplication for attention. It was observed that a huge reduction on ATTE for CaiT (over 56%) when training in ColabPro+. This huge reduction was not as apparent on the other architectures. Nevertheless, CaiT models required more resources and longer training time that all networks in spite of having less parameters than ResNet-50 architecture. Although ResNet-50 and ResNet-50r have more parameters than the other three architectures, the only architecture that trained faster than these two DL networks was MobileNet-v3. However, with a median of 6 epochs, ResNet-50 not only outperforms MobileNet-v3 in training time but all other networks. On the other hand, Exp-14 and Exp-15 achieved the best ranking for number of training epochs accounting for 6 followed by Exp-05.

ResNet-50 and ResNet-50r reached the top ranks for the maximum accuracy during validation while the top ranks by experiment were given to Exp-16 to Exp-18. Exp-17 obtained the highest rank for the maximum validation accuracy. All networks at their best validation accuracy allowed lower accuracy bounds greater from 98.73%.

### 3.5. Non-Parametric Ranks Comparisons

**Figure 7:**
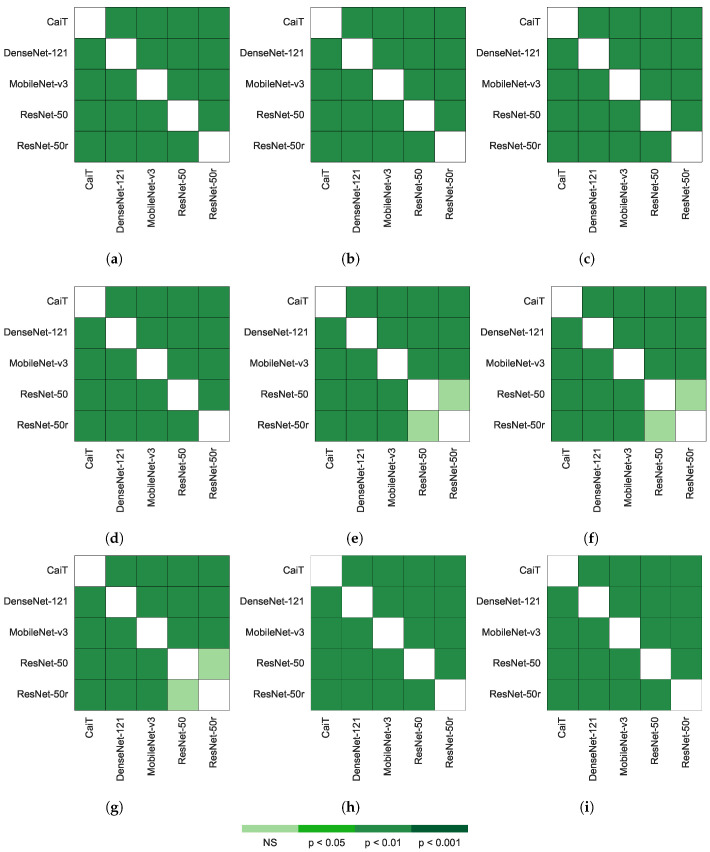
Nemenyi post hoc rankings pairwise of the by network for each of the performance metrics. The comparison was done for all metrics, the maximum accuracy during training and the epoch at which the maximum accuracy was reached. The comparison carried out after 1000 bootstrap cycles. (**a**) Accuracy. (**b**) Balanced accuracy, (**c**) *F*_1_ score, (**d**) *F*_2_ score, (**e**) Matthew’s correlation coefficient. (**f**) Sensitivity. (**g**) Specificity. (**h**) Maximum validation accuracy. (**i**) Number of epochs required to reach the maximum validation accuracy. NS stands for no significant difference.

The results of the Friedman test suggested significant differences among the average ranks of the networks and experiments (*p*-value <0.001). Therefore, the pairwise Friedman–Nemenyi multiple comparisons by network and experiment for all performance metrics was the next step. Figure 7 shows that there was no significant difference on the performance ResNet-50 and ResNet-50r models for the *MCC*, *Sens* and *Spec*. The rest of the architectures performed significantly different to each other for all metrics (*p*-value <0.01). For the maximum accuracy and the number of epochs, all architectures performed significantly different (*p*-value <0.01). This suggest that ResNet-50 networks train faster than the other networks (Table 12).

**Figure 8:**
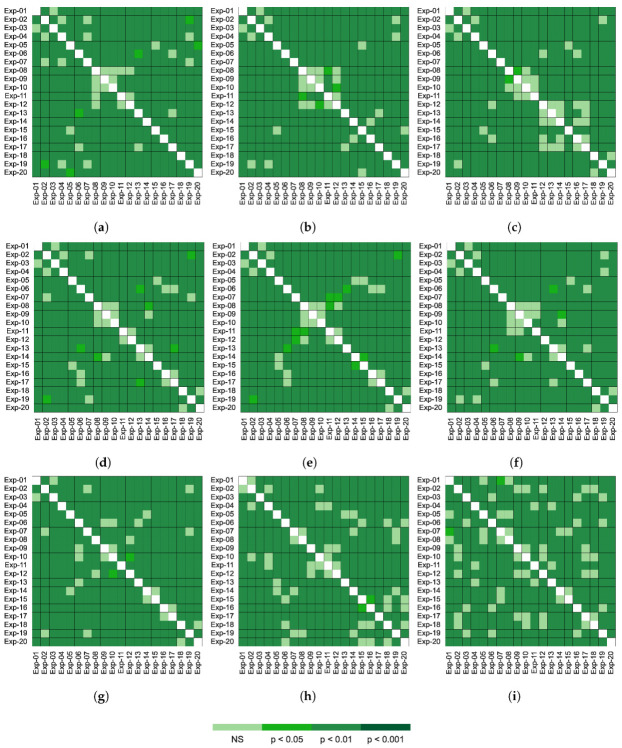
Nemenyi post hoc rankings pairwise comparison for each experiment for all performance metrics after 1000 bootstrap cycles. The probability bar shows the (**a**) Accuracy, (**b**) Balanced accuracy, (**c**) *F*_1_ score, (**d**) *F*_2_ score, (**e**) Matthew’s correlation coefficient. (**f**) Sensitivity. (**g**) Specificity. (**h**) Maximum validation accuracy. (**i**) Number of epochs required to reach the maximum validation accuracy. The NS stands for no significant difference.

Figure 8 shows the Nemenyi test results by experiment for all the performance metrics. Exp-18, Exp-20 obtained the highest ranks for all metrics (Table 10). The two experiments showed no significant difference in the *F*_1_, *F*_2_, *MCC*, *Sens* and *Spec*. Consequently, the two experiments show no statistical difference for the maximum validation accuracy. The Nemenyi test also determined that there was no significant difference in the number of the training epochs required by Exp-14 and Exp-15 to achieved the maximum validation accuracy (top ranks for the number of epochs in Table 13).

In addition, Exp-05 showed no significant difference to Exp-14 and Exp-15 for the *F*_1_ and *MCC*. It can be noted that Exp-19 which was the under performer model based on ResNet-50r has similar predictive power for *BA*, *F*_1_, *Sens* and *Spec* than from Exp-02.

The post hoc tests confirm with 95.0% confidence that in general ResNet-50 based models have a significantly higher performance in the classification of COVID-19 than the other architectures. ResNet-50 architecture not only outperformed the other networks in all metrics but it also make a more effective use of resources by utilising less training time. ResNet-50r models optimized with AdamW outperform all models configurations in all metrics (Exp-18 and Exp-20). However, it requires longer training time, this may be due to the size of the kernel generating more convolutions therefore increasing the number of trainable parameters (Table 1).

The work presented here shows that CaiT performs better when optimising the objective with AdamW (Exp-02 and Exp-04), DenseNet when optimising CE with Adam (Exp-05), MobileNet-v3 optimising CE with Adam or AdamW (Exp-9 and Exp-10). Whereas ResNet-50 performs better when minimising wCE with Adam (Exp-15) and finally ResNet-50r minimising CE with AdamW (Exp-17).

Kernels are small learnable filters that convolve along the depth of an image producing a feature map [82]. It can be observed that the resized kernel on ResNet-50 has a positive effect on experiments Exp-18 and Exp-20 which are the counterpart of Exp-14 and Exp-16. It is possible to attribute this to the fact that the kernel was able to generate feature maps at greater detail for images that might have only small or occluded areas with infection. ResNet-50 is pretrained on the ImageNet dataset which has 1000 object classes of regular size. Whilst identifying a large number of classes represents a challenge in itself, in the medical field to be able to identify small areas of concern is critical for diagnosis. The scope of this work was limited to evaluate the performance of the models by network and experiment after eight rounds of epochs training. Alternatively, from this study of the maximum validation accuracy and the number of epochs by experiment, the post hoc test shows that there is a potential for improvement in the performance of DenseNet-121, MobileNet-v3, ResNet-50 and ResNet-50r. The performance of Exp-06, Exp-12 and Exp-17 can be evaluated after seven rounds of epochs training. Meanwhile the performance of Exp-16 can be measured after six rounds of epochs training.

### 3.6. Limitations of the Present Work

The methodology describe in this paper has several limitations. First, the number of deep learning architectures that was compared was limited. This was a choice as the main objective is not a through comparison of all possible architectures, but rather to present a methodology through which these can be compared with statistical non-parametric tests. Still, a variety of architectures with some of the most recent ones at the time of writing was selected. Second, the data considered for this work were 2D images and not the 3D datasets that can be obtained directly from CT scanners. The authors did not have access to these datasets. Third, this work considered classification of images, but did not extended to segmentation [34], localisation [83], assessment of severity or evolution of the disease [37]. As previously mentioned, one objective was to present a methodology for comparison. Finally, this work is not ready to be deployed in a clinical setting. It is hoped that the methodology here described will help the comparisons of future works and when a methodology is to be deployed clinically, a thorough and fair comparison such as the ones suggested here will be performed.

## 4. Conclusions

In this work, public datasets of chest CT scans were collated and analysed with five AI techniques which were capable to distinguish between positive cases of COVID-19, community-acquired pneumonia and healthy individuals. All the deep learning models were trained and their performance was evaluated with different metrics: accuracy, balanced accuracy, *F*_1_ and *F*_2_ score, *MCC*, sensitivity and specificity. Non-parametric statistics were applied, starting from bootstrapping to obtain confidence intervals, followed with the comparison of the models by using the Friedman test and the Nemenyi pairwise post hoc test. It can be concluded with statistical confidence that the ResNet-50 architectures are robust to classify COVID-19 in a multi-class set-up. ResNet-50 models achieved performances over 98% in all metrics and outperformed MobileNet-v3, DenseNet-121 and CaiT. In the particular case, ResNet-50r which is a modified version of ResNet-50 was shown to be the best classifier when optimising either CE or wCE (Exp-18 and Exp-20) with AdamW. In these conditions, confidence intervals of 99.24% to 99.41%, 99.23% to 99.41%, and 98.48% to 98.86%, were obtained for the *BA*, *F*_1_, *MCC*, respectively. Whilst the metrics of most experiments were high, the rankings after thousands of bootstrap repetitions were more discriminatory and placed ResNet-50r with AdamW in the top place. On the other hand, the CaiT architectures had the lowest rankings. One important observation was that the results suggest that less complex architectures can outperform more complex network architectures in the detection of COVID-19 in a multi-class setup. In general, ResNet-50 showed to be more robust to changes achieving the top ranks in all metrics. With exception of Exp-05, Exp-06, Exp-19, it was observed from Table 10 that Exp-13 to Exp-20 ranked better than experiments Exp-01 to Exp-12 (i.e., those not using ResNet). This study was not aimed to provide causal inference about the reason why ResNet-50 and ResNet-50r networks achieved better results. However, it can be assumed that there was positive interaction between the hyper-parameter selection and experimental setup.

## Figures and Tables

**Table 1:** Architectures and their trainable parameters per architecture considered for the classification of COVID-19 in CT chest scans (3 classes).

Architecture	Parameters	Inference (ms)	Operation	Memory	Year
CaiT	11,763,843	CPU: 75.522 GPU: 57.952	attention matrix multiplication	1.02 Gb	2021
DenseNet-121	6,956,931	CPU: 49.303 GPU: 41.284	convolutions	689 Mb	2017
MobileNet-v3	4,205,875	CPU: 22.692 GPU: 11.40	convolutions	269.01 Mb	2019
ResNet-50	23,514,179	CPU: 42.971 GPU: 40.537	convolutions batch norm	678.85 Mb	2015
ResNet-50r	23,509,571	CPU: 45.068 GPU: 42.788	convolutions batch norm	731.66 Mb	2015

**Table 2:** Class distribution in the training, validation and test for Non-COVID, COVID and Community-acquired pneumonia (CAP).

Class	Training	Validation	Test	Total
COVID-19	6074	759	760	7593
Non-COVID	5514	689	690	6893
CAP	2357	295	294	2946

**Table 3:** Experimental design for the comparison of deep learning networks for the classification of COVID-19 in a multi-class setup. All experiments were trained in batches of 8 images, for 8 epochs and learning rate of 0.00002. CaiT: CaiT-24-XXS-224, MobileNet-v3: MobileNet-v3-large. ResNet50r: ResNet-50 with the first kernel resized from 7×7 to 5×5. CE: Cross Entropy, wCE: weighted Cross Entropy.

Experiment	Architecture	Loss	Optimizer
Exp-01	CaiT	CE	Adam
Exp-02	CaiT	CE	AdamW
Exp-03	CaiT	wCE	Adam
Exp-04	CaiT	wCE	AdamW
Exp-05	DenseNet-121	CE	Adam
Exp-06	DenseNet-121	CE	AdamW
Exp-07	DenseNet-121	wCE	Adam
Exp-08	DenseNet-121	wCE	AdamW
Exp-09	MobileNet-v3	CE	Adam
Exp-10	MobileNet-v3	CE	AdamW
Exp-11	MobileNet-v3	wCE	Adam
Exp-12	MobileNet-v3	wCE	AdamW
Exp-13	ResNet-50	CE	Adam
Exp-14	ResNet-50	CE	AdamW
Exp-15	ResNet-50	wCE	Adam
Exp-16	ResNet-50	wCE	AdamW
Exp-17	ResNet-50r	CE	Adam
Exp-18	ResNet-50r	CE	AdamW
Exp-19	ResNet-50r	wCE	Adam
Exp-20	ResNet-50r	wCE	AdamW

**Table 4:** Train, validation and test accuracy by architecture before bootstrapping for all architectures. CaiT (Exp-01:Exp-04), DenseNet-121 (Exp-05:Exp-08), MobileNet-v3-large (Exp-09:Exp-12), ResNet-50 (Exp-13:Exp-16), ResNet-50r (Exp-17:Exp-20). Best results are highlighted in bold.

Architecture	Train Accuracy	Validation Accuracy	Test Accuracy
CaiT	99.23 ± 0.79	98.43 ± 0.64	98.55 ± 0.54
DenseNet-121	**99.42** ± 0.10	99.07 ± 0.29	98.89 ± 0.40
MobileNet-v3	98.42 ± 0.58	98.65 ± 0.46	98.86 ± 0.33
ResNet-50	99.36 ± 0.10	**99.16** ± 0.32	**99.02** ± 0.22
ResNet-50r	99.20 ± 0.14	99.10 ± 0.40	98.99 ± 0.32

**Table 5:** Train, validation and test average accuracy and standard deviation by experiment before bootstrapping. Best top three average accuracy results are highlighted in bold.

Experiment	Train Accuracy	Validation Accuracy	Test Accuracy
Exp-01	99.38 ± 0.06	98.25 ± 0.60	98.47 ± 0.41
Exp-02	99.38 ± 0.12	98.70 ± 0.43	98.64 ± 0.44
Exp-03	98.84 ± 1.56	98.35 ± 0.73	98.44 ± 0.52
Exp-04	99.31 ± 0.13	98.41 ± 0.76	98.64 ± 0.76
Exp-05	**99.45** ± 0.06	99.10 ± 0.20	**99.08** ± 0.17
Exp-06	**99.44** ± 0.06	99.13 ± 0.22	98.97 ± 0.30
Exp-07	99.40 ± 0.12	99.01 ± 0.34	98.67 ± 0.55
Exp-08	99.38 ± 0.12	99.03 ± 0.40	98.86 ± 0.42
Exp-09	98.61 ± 0.09	98.77 ± 0.36	98.89 ± 0.22
Exp-10	98.24 ± 1.17	98.57 ± 0.71	98.87 ± 0.54
Exp-11	98.41 ± 0.09	98.56 ± 0.43	98.83 ± 0.32
Exp-12	98.44 ± 0.05	98.70 ± 0.22	98.84 ± 0.18
Exp-13	**99.42** ± 0.07	**99.17** ± 0.29	98.94 ± 0.33
Exp-14	99.38 ± 0.08	99.15 ± 0.30	99.04 ± 0.18
Exp-15	99.34 ± 0.06	**99.18** ± 0.41	**99.08** ± 0.16
Exp-16	99.31 ± 0.15	99.14 ± 0.31	99.01 ± 0.19
Exp-17	99.22 ± 0.17	**99.19** ± 0.30	98.97 ± 0.34
Exp-18	99.22 ± 0.15	**99.19** ± 0.35	**99.16** ± 0.19
Exp-19	99.17 ± 0.10	98.94 ± 0.53	98.71 ± 0.29
Exp-20	99.20 ± 0.15	99.08 ± 0.39	**99.13** ± 0.23

**Table 6:** Evaluation metrics by architecture before bootstrapping. Best results are highlighted in bold.

Architecture	Acc	BA	*F* ${}_{1}$	*F* ${}_{2}$
CaiT	98.55 ± 0.54	98.71 ± 0.66	98.70 ± 0.63	98.70 ± 0.65
DenseNet-121	98.89 ± 0.40	99.10 ± 0.34	99.10 ± 0.34	99.08 ± 0.37
MobileNet-v3	98.86 ± 0.33	99.06 ± 0.30	99.06 ± 0.29	99.06 ± 0.29
ResNet-50	**99.02** ± 0.22	**99.19** ± 0.20	99.15 ± 0.22	99.16 ± 0.22
ResNet-50r	98.99 ± 0.32	99.16 ± 0.32	**99.16** ± 0.30	**99.18** ± 0.31
Architecture	MCC	Sens	Spec	
CaiT	97.64 ± 0.86	98.72 ± 0.67	99.17 ± 0.28	
DenseNet-121	98.27 ± 0.60	99.11 ± 0.34	99.36 ± 0.23	
MobileNet-v3	98.18 ± 0.52	99.08 ± 0.29	99.35 ± 0.25	
ResNet-50	**98.42** ± 0.37	**99.17** ± 0.22	**99.43** ± 0.13	
ResNet-50r	98.41 ± 0.51	**99.17** ± 0.32	**99.43** ± 0.18	

**Table 7:** Evaluation metrics by experiment before bootstrapping. Average and standard deviation per each metric are given in percentage. The best top three values per metric are highlighted in bold.

Experiments	*Acc*	*BA*	*F*_1_	*F*_2_	*MCC*	*Sens*	*Spec*
Exp-01	98.47 ± 0.41	98.61 ± 0.40	98.65 ± 0.36	98.62 ± 0.40	97.52 ± 0.65	98.63 ± 0.41	99.12 ± 0.23
Exp-02	98.64 ± 0.44	98.89 ± 0.36	98.83 ± 0.43	98.86 ± 0.39	97.84 ± 0.69	98.88 ± 0.36	99.25 ± 0.23
Exp-03	98.44 ± 0.52	98.52 ± 1.06	98.62 ± 0.76	98.56 ± 0.95	97.52 ± 0.83	98.56 ± 1.09	99.12 ± 0.30
Exp-04	98.64 ± 0.76	98.81 ± 0.63	98.71 ± 0.90	98.77 ± 0.74	97.66 ± 1.23	98.81 ± 0.63	99.19 ± 0.38
Exp-05	99.08 ± 0.17	99.23 ± 0.15	99.22 ± 0.21	99.22 ± 0.17	98.51 ± 0.28	99.23 ± 0.15	99.47 ± 0.09
Exp-06	98.97 ± 0.30	99.16 ± 0.25	99.14 ± 0.26	99.15 ± 0.26	98.35 ± 0.48	99.16 ± 0.25	99.38 ± 0.16
Exp-07	98.67 ± 0.55	98.96 ± 0.50	98.97 ± 0.46	98.86 ± 0.53	98.04 ± 0.83	98.97 ± 0.51	99.25 ± 0.32
Exp-08	98.86 ± 0.42	99.05 ± 0.36	99.08 ± 0.36	99.09 ± 0.37	98.17 ± 0.65	99.09 ± 0.35	99.34 ± 0.24
Exp-09	98.89 ± 0.22	99.08 ± 0.19	99.06 ± 0.16	99.10 ± 0.20	98.22 ± 0.34	99.10 ± 0.21	99.37 ± 0.13
Exp-10	98.87 ± 0.54	99.07 ± 0.48	99.03 ± 0.46	99.07 ± 0.47	98.20 ± 0.85	99.09 ± 0.48	99.36 ± 0.34
Exp-11	98.83 ± 0.32	99.03 ± 0.30	99.04 ± 0.21	99.04 ± 0.25	98.14 ± 0.50	99.08 ± 0.23	99.30 ± 0.34
Exp-12	98.84 ± 0.18	99.06 ± 0.15	99.12 ± 0.29	99.04 ± 0.17	98.15 ± 0.28	99.05 ± 0.16	99.35 ± 0.12
Exp-13	98.94 ± 0.33	99.13 ± 0.29	99.11 ± 0.31	99.12 ± 0.29	98.30 ± 0.55	99.13 ± 0.28	99.39 ± 0.19
Exp-14	99.04 ± 0.18	99.19 ± 0.20	99.11 ± 0.22	99.12 ± 0.25	98.48 ± 0.30	99.12 ± 0.25	99.47 ± 0.10
Exp-15	99.08 ± 0.16	99.26 ± 0.14	99.23 ± 0.13	99.22 ± 0.14	98.53 ± 0.26	99.23 ± 0.15	99.46 ± 0.11
Exp-16	99.01 ± 0.19	99.18 ± 0.17	99.13 ± 0.17	99.16 ± 0.16	98.38 ± 0.31	99.20 ± 0.21	99.42 ± 0.11
Exp-17	98.97 ± 0.34	99.13 ± 0.37	99.11 ± 0.35	99.15 ± 0.34	98.36 ± 0.55	99.15 ± 0.37	99.43 ± 0.19
Exp-18	**99.16** ± 0.19	**99.32** ± 0.15	**99.32** ± 0.15	**99.32** ± 0.15	**98.66** ± 0.31	**99.32** ± 0.15	**99.52** ± 0.11
Exp-19	98.71 ± 0.29	98.90 ± 0.35	98.90 ± 0.27	98.92 ± 0.34	97.94 ± 0.46	98.90 ± 0.35	99.27 ± 0.17
Exp-20	**99.13** ± 0.23	**99.28** ± 0.20	**99.30** ± 0.21	**99.31** ± 0.20	**98.66** ± 0.35	**99.31** ± 0.20	**99.51** ± 0.13

**Table 8:** Ranks and medians by architecture after 1000 bootstrapping cycles. Best results are highlighted in bold.

Architecture	Rank						
	*Acc*	*BA*	*F*_1_	*F*_2_	*MCC*	*Sens*	*Spec*
CaiT	5.00	5.00	5.00	5.00	5.00	5.00	5.00
DenseNet-121	3.20	2.98	2.82	3.18	3.05	2.92	3.30
MobileNet-v3	3.63	3.62	3.58	3.50	3.72	3.54	3.56
ResNet-50	**1.40**	**1.38**	1.92	1.84	**1.53**	**1.75**	**1.51**
ResNet-50r	1.77	2.02	**1.68**	**1.47**	1.71	1.80	1.63
Architecture	Median						
	*Acc*	*BA*	*F*_1_	*F*_2_	*MCC*	*Sens*	*Spec*
CaiT	98.56	98.72	98.71	98.72	97.65	98.73	99.17
DenseNet-121	98.89	99.10	99.11	99.08	98.27	99.12	99.36
MobileNet-v3-large	98.86	99.07	99.07	99.06	98.19	99.08	99.35
ResNet-50	**99.02**	**99.19**	99.14	99.15	**98.42**	99.17	**99.43**
ResNet-50r	98.99	99.16	**99.16**	**99.18**	98.41	**99.18**	**99.43**

**Table 9:** Confidence intervals by architecture after 1000 bootstrapping cycles with α=0.05. Best results are highlighted in bold.

Architecture	Accuracy	*BA*	*F*_1_	*F*_2_
CaiT	98.37–98.71	98.49–98.90	98.50–98.87	98.49–98.88
DenseNet-121	98.76–99.00	98.99–99.20	99.00–99.20	98.96–99.19
MobileNet-v3 ^1^	98.75–98.95	98.97–99.15	98.96–99.15	98.97–99.15
ResNet-50	**98.95–99.09**	**99.13–99.25**	**99.07–99.21**	**99.09–99.22**
ResNet-50r	**98.90–99.08**	**99.05–99.25**	**99.07–99.25**	**99.09–99.26**
Architecture	*MCC*	*Sens*	*Spec*	
CaiT	97.36–97.88	98.48–98.90	99.08–99.25	
DenseNet-121	98.07–98.44	99.00–99.21	99.29–99.43	
MobileNet-v3	98.01–98.33	98.98–99.16	99.26–99.42	
ResNet-50	**98.31–98.53**	**99.10–99.24**	**99.39–99.47**	
ResNet-50r	**98.25–98.57**	**99.07–99.26**	**99.38–99.48**	

^1^ MobileNet-v3-large is the full named of the architecture.

**Table 10:** Ranks by experiment after 1000 bootstraps cycles. Best results are highlighted in bold. Experiments 18 and 20 correspond to ResNet-50r with AdamW, CE (18) and wCE (20).

Experiment	Rank							Median						
	*Acc*	*BA*	*F*_1_	*F*_2_	*MCC*	*Sens*	*Spec*	*Acc*	*BA*	*F*_1_	*F*_2_	*MCC*	*Sens*	*Spec*
Exp-01	18.70	19.14	18.89	18.96	18.84	19.05	18.78	98.46	98.61	98.65	98.62	97.52	98.62	99.12
Exp-02	16.51	16.03	16.67	16.11	16.49	16.40	15.69	98.64	98.90	98.84	98.87	97.85	98.89	99.26
Exp-03	18.90	18.96	18.59	18.75	18.85	18.75	18.68	98.45	98.54	98.64	98.58	97.53	98.57	99.12
Exp-04	15.82	16.51	17.15	16.96	17.30	16.84	16.79	98.64	98.85	98.74	98.78	97.70	98.82	99.20
Exp-05	3.90	4.50	4.40	4.70	4.64	4.78	4.20	99.08	99.24	99.23	99.22	98.51	99.23	99.47
Exp-06	7.92	7.87	7.77	7.69	8.15	7.84	9.88	98.97	99.16	99.14	99.15	98.35	99.16	99.38
Exp-07	15.98	14.18	13.62	15.75	13.55	13.83	15.41	98.67	98.97	98.98	98.87	98.07	98.98	99.25
Exp-08	11.58	11.50	10.42	10.02	11.57	10.59	11.75	98.86	99.06	99.08	99.09	98.18	99.09	99.35
Exp-09	10.88	10.92	11.46	9.84	10.86	10.48	10.23	98.89	99.08	99.06	99.10	98.23	99.10	99.38
Exp-10	10.82	10.72	11.36	10.22	10.88	9.93	10.57	98.89	99.08	99.05	99.08	98.22	99.11	99.37
Exp-11	12.44	12.49	11.89	12.26	12.60	11.18	13.31	98.84	99.04	99.05	99.04	98.14	99.08	99.31
Exp-12	12.38	11.70	8.64	12.45	12.51	12.56	11.58	98.84	99.07	99.11	99.04	98.15	99.05	99.35
Exp-13	8.98	9.10	9.23	8.68	9.19	8.85	8.97	98.95	99.13	99.11	99.13	98.31	99.14	99.40
Exp-14	5.49	6.44	8.93	9.03	5.24	9.51	4.49	99.05	99.20	99.12	99.12	98.48	99.12	99.47
Exp-15	4.29	3.76	4.11	4.57	4.29	4.76	5.33	99.08	99.26	99.23	99.22	98.52	99.23	99.45
Exp-16	6.87	6.66	8.05	7.37	7.58	6.11	7.65	99.01	99.19	99.14	99.16	98.38	99.20	99.42
Exp-17	8.23	8.95	8.98	7.68	7.85	8.31	7.07	98.97	99.14	99.12	99.15	98.36	99.16	99.43
Exp-18	**1.84**	**1.66**	**1.70**	**1.69**	**2.06**	**1.87**	**1.70**	99.16	99.32	99.32	99.32	98.66	99.32	99.53
Exp-19	15.55	15.90	15.81	15.07	15.52	16.09	15.30	98.71	98.90	98.90	98.93	97.95	98.90	99.27
Exp-20	**2.92**	**3.03**	**2.32**	**2.20**	**2.03**	**2.28**	**2.62**	99.12	99.28	99.30	99.31	98.66	99.31	99.50

**Table 11:** Confidence interval by experiment for *Acc*, *BA*, *F*_1_, *F*_2_, *MCC*, *Sens* and *Spec* after 1000 bootstrapping cycles with α=0.05. Best results are highlighted in bold. Experiment 18 corresponds to ResNet-50r with AdamW and CE.

Experiment	*Acc*	*BA*	*F*_1_	*F*_2_	*MCC*	*Sens*	*Spec*
Exp-01	98.24–98.71	98.36–98.85	98.45–98.86	98.39–98.85	97.14–97.91	98.40–98.88	98.99–99.25
Exp-02	98.38–98.87	98.67–99.10	98.56–99.06	98.62–99.08	97.40–98.21	98.67–99.08	99.09–99.37
Exp-03	98.09–98.67	97.82–98.92	98.13–98.92	97.91–98.92	96.96–97.88	97.83–99.00	98.92–99.25
Exp-04	98.12–98.94	98.42–99.12	98.13–99.11	98.26–99.11	96.89–98.24	98.39–99.12	98.96–99.38
Exp-05	98.99–99.18	99.15–99.32	99.08–99.34	99.11–99.31	98.35–98.66	99.15–99.32	99.42–99.52
Exp-06	98.80–99.14	99.02–99.30	98.99–99.29	99.01–99.30	98.08–98.63	99.02–99.31	99.28–99.47
Exp-07	98.33–98.95	98.64–99.21	98.68–99.20	98.55–99.17	97.48–98.47	98.64–99.25	99.06–99.42
Exp-08	98.61–99.10	98.85–99.26	98.85–99.27	98.87–99.29	97.79–98.54	98.88–99.28	99.20–99.47
Exp-09	98.75–99.02	98.97–99.19	98.96–99.16	98.98–99.20	98.02–98.41	98.96–99.21	99.29–99.45
Exp-10	98.50–99.14	98.74–99.32	98.72–99.26	98.77–99.31	97.69–98.62	98.79–99.34	99.14–99.53
Exp-11	98.64–99.00	98.84–99.19	98.91–99.15	98.89–99.18	97.82–98.40	98.94–99.21	99.07–99.46
Exp-12	98.73–98.94	98.97–99.14	98.99–99.31	98.94–99.13	97.99–98.30	98.95–99.14	99.28–99.41
Exp-13	98.74–99.13	98.95–99.29	98.91–99.28	98.94–99.29	97.95–98.61	98.96–99.29	99.29–99.50
Exp-14	98.93–99.14	99.06–99.30	98.97–99.24	98.95–99.25	98.29–98.63	98.96–99.25	99.41–99.52
Exp-15	98.99–99.18	99.17–99.34	99.16–99.30	99.14–99.31	98.38–98.68	99.15–99.32	99.39–99.52
Exp-16	98.90–99.11	99.08–99.27	99.03–99.22	99.06–99.24	98.17–98.56	99.08–99.33	99.35–99.49
Exp-17	98.75–99.16	98.90–99.33	98.89–99.30	98.94–99.35	98.05–98.69	98.91–99.35	99.31–99.53
**Exp-18**	**99.06–99.28**	**99.24–99.41**	**99.23–99.41**	**99.24–99.41**	**98.48–98.86**	**99.23–99.41**	**99.47–99.59**
Exp-19	98.55–98.87	98.70–99.08	98.73–99.06	98.72–99.11	97.69–98.22	98.68–99.08	99.16–99.36
Exp-20	98.99–99.26	99.17–99.40	99.18–99.42	99.19–99.43	98.46–98.87	99.20–99.43	99.44–99.58

**Table 12:** Ranking of the maximum training accuracy and training epochs, medians and confidence intervals (CI) by architecture with α=0.05 and Average training time per epoch (ATTE). Best results are highlighted in bold.

Architecture	Max Accuracy			Epochs			
	Rank	Median	CI	Rank	Median	CI	ATTE (s)
CaiT	4.92	98.82	98.73–98.91	3.38	8	5–8	503.66 220.57 ^1^
DenseNet-121	2.78	99.31	99.26–99.35	2.19	7	5–7	177.08
MobileNet-v3	4.08	98.92	98.82–99.02	3.14	7	7–8	87.60
ResNet-50	1.85	99.34	99.30–99.38	**1.93**	**6**	**6–8**	100.31
ResNet-50r	**1.37**	**99.36**	**99.30–99.42**	4.36	8	6–8	106.05

^1^ Running in Google Colab Pro+.

**Table 13:** Ranking of the maximum training accuracy and training epochs with medians by experiment. Best results are highlighted in bold.

Experiment	Max Accuracy		Epochs	
	Rank	Median	Rank	Median
Exp-01	17.59	98.80	8.14	5
Exp-02	17.05	98.84	11.39	7
Exp-03	19.05	98.76	9.79	7
Exp-04	16.42	98.88	13.45	8
Exp-05	7.55	99.32	**8.09**	6
Exp-06	5.69	99.35	9.31	7
Exp-07	9.21	99.30	7.18	6
Exp-08	9.69	99.28	8.11	6
Exp-09	15.10	98.93	12.28	7
Exp-10	16.41	98.88	11.75	7
Exp-11	15.53	98.91	13.64	7
Exp-12	14.76	98.95	11.61	7
Exp-13	6.98	99.33	14.11	8
Exp-14	8.38	99.31	**6.58**	6
Exp-15	5.25	99.36	**6.65**	6
Exp-16	**4.27**	99.37	9.95	6
Exp-17	**1.91**	99.43	12.13	7
Exp-18	**4.98**	99.36	11.20	8
Exp-19	9.16	99.28	9.28	6
Exp-20	5.02	99.37	15.36	8

## Data Availability

Datasets are available at: https://www.kaggle.com/datasets/maedemaftouni/large-covid19-ct-slice-dataset (accessed on 3 August 2022) https://data.mendeley.com/datasets/3y55vgckg6/2 (accessed on 3 August 2022).

## Abbreviations

The following abbreviations are used in this manuscript:

*Acc*	Accuracy
ATTE	Average training time per epoch
*BA*	Balanced accuracy
CAP	Community acquired pneumonia
CE	Cross entropy
CT	Computed tomography
*MCC*	Mathew’s correlation coefficient
*Sens*	Sensitivity
*Spec*	Specificity
Val	Validation
wCE	Weighted cross entropy
